# Development of a Genoserotyping Method for *Salmonella* Infantis Detection on the Basis of Pangenome Analysis

**DOI:** 10.3390/microorganisms9010067

**Published:** 2020-12-29

**Authors:** Seung-Min Yang, Jiwon Baek, Eiseul Kim, Hyeon-Be Kim, Seyoung Ko, Donghyuk Kim, Hyunjin Yoon, Hae-Yeong Kim

**Affiliations:** 1 Institute of Life Sciences & Resources and Department of Food Science and Biotechnology, Kyung Hee University, Yongin 17104, Korea; ysm9284@gmail.com (S.-M.Y.); 20080600@hanmail.net (E.K.); com0611@naver.com (H.-B.K.); 2 Department of Molecular Science and Technology, Ajou University, Suwon 16499, Korea; qorwldnjs78@ajou.ac.kr; 3 School of Life Sciences, Ulsan National Institute of Science and Technology (UNIST), Ulsan 44919, Korea; sierrayk@gmail.com (S.K.); smallvug@gmail.com (D.K.); 4 Department of Applied Chemistry and Biological Engineering, Ajou University, Suwon 16499, Korea

**Keywords:** *Salmonella* Infantis, genoserotyping, pangenome, real-time PCR

## Abstract

In recent years, *Salmonella* Infantis has become a predominant serovariant in clinical and poultry isolates, thereby imposing a substantial economic burden on both public health and the livestock industry. With the aim of coping with the steep increase in serovar Infantis prevalence, a polymerase chain reaction (PCR)-based rapid and accurate diagnostic assay was developed in this study through pangenome profiling of 60 *Salmonella* serovars. A gene marker, SIN_02055, was identified, which is present in the *S*. Infantis genome but not in the pangenome of the other serovars. Primers specific to SIN_02055 were used to accurately detect serovar Infantis, and to successfully differentiate Infantis from the other 59 serovars in real-time PCR with a R^2^ of 0.999 and an efficiency of 95.76%. The developed method was applied to 54 *Salmonella* strains belonging to eight dominant serovars, and distinguished Infantis from the other seven serovars with an accuracy of 100%. The diagnostic primer set also did not show false positive amplification with 32 strains from eight non-*Salmonella* bacterial species. This cost-effective and rapid method can be considered an alternative to the classic serotyping using antisera.

## 1. Introduction

*Salmonella* is a leading cause of foodborne illnesses, and generally provokes self-limiting gastroenteritis with symptoms of diarrhea, abdominal cramps, fever, and vomiting. Nonetheless, it is often invasive and causes fatal bacteremia and extraintestinal colonization in young children, the elderly, and immunocompromised patients [1,2]. The main sources of human infection are contaminated animal products, especially poultry products. For several decades, *Salmonella* Enteritidis and *S*. Typhimurium have been regarded as the most notorious serovars responsible for human infections [3,4]. On the other hand, recent surveillance reports indicate that the prevalence of these serovars has declined, while *S.* Infantis has become the predominant serovar in clinical and poultry isolates [5,6,7]. The current epidemiological trends in causative serovars will presumably continue for some time, owing to gradual implementation of eradication measures such as poultry vaccination against *S*. Enteritidis and *S*. Typhimurium [5,8]. Thus, *S.* Infantis is expected to rank among the most important serovars associated with public health programs.

The *Salmonella* genus consists of two species, *S. enterica* and *S. bongori*, and serotyping based on the antigenic surface structures of lipopolysaccharide (O antigen) and flagellar proteins (H1 and H2 antigens) further subdivides this genus into over 2600 serovars [9]. Therefore, in terms of an epidemiological approach, *Salmonella* serotyping is a prerequisite for detecting the emergence and prevalence of pathogenic serovars in various geographic regions. Additionally, surveillance monitoring of serovars circulating on farms enables effective timely control, thereby preventing the dissemination of specific serovars into the food chain. Nevertheless, the results of conventional serotyping through antiserum agglutination with surface antigens are subject to misinterpretation because of incomplete expression and presentation of antigenic determinants targeted by the antisera [10]. The necessity of expertise and a comprehensive set of antisera is also a hindrance to the implementation of general laboratory diagnostic testing for *Salmonella*. Above all, the current time-consuming low-throughput procedure is the main disadvantage of the classic serotyping.

As alternatives to conventional serotyping, diverse molecular approaches independent of antigen production have been attempted, including pulsed-field gel electrophoresis [11], 16S to 23S ribosomal-RNA spacer restriction fragment length polymorphism [12], and multilocus sequence typing [13]. Molecular methods involving DNA markers within the genes responsible for O and/or H antigen production have also been devised for representative serovars [8,14]. Nonetheless, these methods do not always guarantee high enough resolution performance on discrimination of serovars. Whole-genome sequencing provides comprehensive genetic information regarding bacterial species, virulence, and evolutionary history, and this versatile tool is now replacing current molecular serotyping methods [15]. For example, web-based tools such as SeqSero and *Salmonella* In Silico Typing Resource (SISTR) can identify the serotype of a causative agent rapidly and accurately by aligning the whole-genome sequences with available genome sequence data [16,17]. In this context, whole-genome sequencing is extensively exploited to track the transmission path of causative disease agents and define epidemiological significance of infectious agents [16]. Keeping pace with advanced sequencing technologies and sophisticated bioinformatic tools, genoserotyping methods that can determine *Salmonella* serovars at low cost and high resolution without expertise have emerged due to the availability of whole-genome sequences [18,19]. In the present study, the core genome conserved in *S.* Infantis strains was compared with the pangenome of 555 *Salmonella* strains from 59 serovars other than Infantis, and a DNA-based diagnostic assay targeting a gene marker specific to serovar Infantis was developed for feasible incorporation into diagnostic laboratory practice.

## 2. Materials and Methods

### 2.1. Bacterial Strains and Culture Conditions

Bacterial strains, including both *Salmonella* (107 strains) and non-*Salmonella* taxa (32 strains), were cultivated in Tryptic Soy broth (TSB, BD Difco Laboratory Inc., Sparks, MD, USA) at 37 °C for 18 h. The bacterial cells were harvested by centrifugation at 13,600× *g* and 4 °C for 10 min, and bacterial genomic DNA was extracted using the DNeasy Blood & Tissue Kit (Qiagen, Hilden, Germany). The concentration and purity of the extracted genomic DNA were measured on a MaestroNano^®^ spectrophotometer (Maestrogen, Las Vegas, NV, USA).

### 2.2. Comparative Genome Analysis

A total of 568 *Salmonella* genome sequences representing 60 serovars were downloaded from the National Center for Biotechnology Information (NCBI), and the corresponding strains are listed in Supplementary Table S1. Gene markers specific to serovar Infantis were identified by means of Bacterial Pan Genome Analysis (BPGA) pipeline v1.3 [20]. The annotated genome sequences were employed to construct a pangenome with a default cutoff of 50% sequence identity via the USEARCH v9.0 clustering algorithm. Candidate marker genes were defined as genes that were conserved in all genome sequences of *S*. Infantis strains but did not share any significant sequence homology with genomes of the other serovars. The 568 genome sequences were compiled into two separate local databases: One included the core genome of serovar Infantis, and the other encompassed the pangenome database for all serovars other than *S*. Infantis. The two genome databases (the core genome and pangenome) were compared to identify the candidate gene markers specific to serovar Infantis. Pangenome analysis was visualized with a microbial pangenomics workflow implemented in Anvi’o v6.1 packages [21]. Briefly, the assembled genomes served as an input file for the workflow to construct Anvi’o genome storage, which was in turn clustered and reassembled based on sequence similarity via the NCBI BLASTp (Protein Basic Local Alignment Search Tool) algorithm and Markov cluster (MCL) algorithm. Next, Python code of anvi-display-pan was used to visualize the pangenome result. A phylogenetic tree based on the pangenome was then constructed according to the pan gene cluster frequencies. Comparative analysis of the loci corresponding to a gene marker was conducted in eight *Salmonella* serovars on Web server SyntTax [22].

### 2.3. Primer Design for a Diagnostic PCR Assay

The retrieved candidate gene markers were then aligned with 57,014,819 bacterial sequences by means of NCBI BLAST, and an Infantis-specific gene marker present only in *S.* Infantis (i.e., not in the other sequences) was identified. The diagnostic primer set for the gene marker was designed using Primer Designer (Scientific and Education Software, NC, USA) with the following criteria: primer length, 18–30 bases; T_m_, 52–58 °C; and GC content, 45–60% [23]. The forward and reverse primer sequences were 5′-GGT CGA GAT GGG TAT GTA GC-3′ and 5′-CAG GAG TTC CTG CGC AAC CA-3′, respectively. The efficacy of the diagnostic-PCR primer set in differentiating *S.* Infantis from other serovars was evaluated using an in silico PCR amplification tool [24]. Genomic loci that could be amplified by the diagnostic PCR primer set were predicted via alignment of the primer sequences with genome sequences of 200 *Salmonella* strains that are highly prevalent or possess antigenic determinants similar to those of *S.* Infantis. In silico PCR conditions were confined to those that yield amplicons shorter than 3000 nucleotides and allow two mismatches, with one of them located at the 3′ end.

### 2.4. Conventional Serotyping through Antisera Agglutination

*Salmonella* serological affiliation was determined using the antigenic formulae used by the World Health Organization Collaborating Centre for Reference and Research on *Salmonella* at the Pasteur Institute, Paris, France (WHO Collaborating Center, 2007) [25]. All *Salmonella* antisera were purchased from BD Difco Laboratory Inc. (Sparks, MD, USA). *Salmonella* strains were cultivated in the brain heart infusion (BHI) medium (BD Difco) or Motility GI medium (BD Difco) for analyses of the somatic O antigen or flagellar H antigens, respectively. For somatic antigen identification, a 10 μL aliquot of antiserum was mixed with a drop of a bacterial suspension on a glass slide. The slide was rocked back and forth for 1 min, and then an agglutination reaction was observed. For flagellar-antigen determination, flagellar phase induction was ensured by motility tests, and flagellar antigens were fixed by treatment with 10 mL of 0.6% formalin for 1 h prior to the reaction with antisera. Aliquots (0.5 mL) of the bacterial suspension were mixed with 0.5 mL of antiserum solutions (serial dilutions) in glass test tubes and were incubated at 50 °C for 1 h. The agglutination reactions were examined within 2 h after incubation at 50 °C. Vi antigen testing was not conducted because of the lack of capsule formation in the tested *Salmonella* strains.

### 2.5. PCR and Real-Time PCR

Regular PCR with the designed primer set was conducted on a thermocycler (Astec, Fukuoka, Japan) under the following conditions: 94 °C for 5 min; 35 cycles of 94 °C for 10 s, 60 °C for 10 s, and 72 °C for 10 s; and 72 °C for 5 min. The reaction mixture (25 µL) contained a DNA template (20 ng), a primer set (400 nM each), and 0.5 U of *Taq* DNA polymerase (TaKaRa Bio Inc., Tokyo, Japan). The PCR products were analyzed using electrophoresis (2% agarose gel) and a UV transilluminator (Vilber Lourmat, Marne La Vallee, France).

Real-time PCR was carried out on a 7500 Real-Time PCR System (Applied Biosystems, Foster City, CA, USA). To test the specificity of the diagnostic primers, the reaction mixture (20 µL) was prepared from 20 ng of a template DNA, 500 nM primer set, 10 µL of the 2× Thunderbird SYBR^®^ qPCR Mix (Toyobo, Osaka, Japan), and distilled water. To evaluate assay accuracy, a standard curve was constructed using serial dilutions (from 20 ng to 2 pg) of the genomic DNA of *S.* Infantis strain CCARM 8283 in triplicate. Real-time PCR was initiated by a 2 min incubation at 95 °C, followed by 30 cycles of 95 °C for 5 s and 60 °C for 30 s. Melting curves were generated under following conditions: 95 °C for 15 s, 60 °C for 1 min, 95 °C for 30 s, and 60 °C for 15 s. The results of the real-time PCR were confirmed in 7500 software v2.3 (Applied Biosystems).

### 2.6. Evaluation of Sensitivity, Specificity, and Accuracy of the Diagnostic Assay

These characteristics of the diagnostic real-time PCR assay were determined using the numbers of true positive (TP), true negative (TN), false negative (FN), and false positive (FP) results: sensitivity = TP/(TP + FN); specificity = TN/(TN + FP); and accuracy = (TP + TN)/(TP + TN + FN + FP).

## 3. Results

### 3.1. Identification of Gene Markers Specific to Serovar Infantis

**Figure 1:**
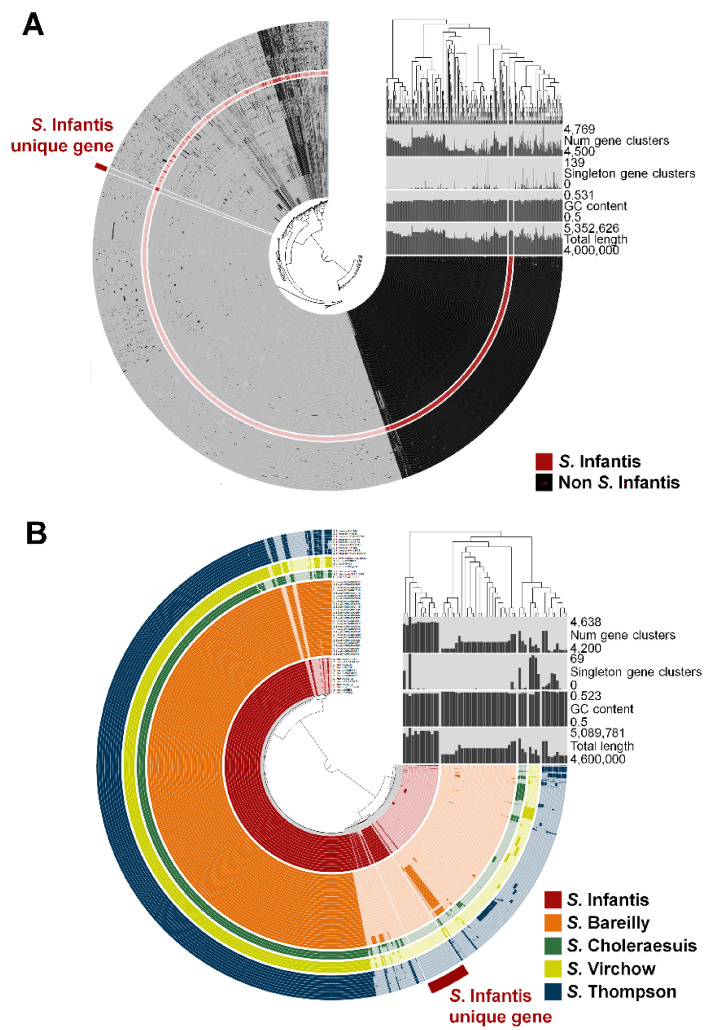
Pangenome distribution of (**A**) 568 *Salmonella* strains of 60 serovars and (**B**) 56 strains of five serovars, namely *S*. Infantis, *S*. Bareilly, *S*. Choleraesuis, *S*. Virchow, and *S*. Thompson. Each arc represents a genome, and each layer displays the pangenome distribution. Black arcs in (**A**) represent the genomes of 555 *Salmonella* strains not belonging to *S*. Infantis. The dark and bright regions of each arc indicate core genome presence and absence, respectively. The red, orange, green, yellow, and blue arcs in (**B**) denote the genomes of *S*. Infantis, *S*. Bareilly, *S*. Choleraesuis, *S*. Virchow, and S. Thompson, respectively.

Genome sequences from 568 *Salmonella* strains representing 60 serovars (Supplementary Table S1) were subjected to pangenome analysis. The 60 serovars encompassed major causative agents of foodborne illnesses, including Enteritidis (81 strains), Typhimurium (51 strains), Newport (12 strains), and Infantis (13 strains). The core genome was determined via 13 complete *S.* Infantis genomes (Supplementary Figure S1), and was compared with the pangenome representing 555 genomes of 59 other serovars (Figure 1A). A total of 66 genes present only in the Infantis core genome (not in the pangenome of the other serovars) were sorted out by comparative analysis, and SIN_02055 in gene *usg* in *S.* Infantis strain 1326/28 [26] was identified as a candidate gene marker of serovar Infantis. The other 65 core genes had orthologues in other bacterial species, and were excluded. There were several serovars that possessed antigenic determinants similar to those of the H antigens of *S.* Infantis. The flagellar apparatus, which is analyzed for differentiation between H antigens, is frail and frequently decomposed, leading to an ambiguous serotyping interpretation [8]. The pangenome analysis included four serovars that are easily confused with Infantis (6,7,14:r:1,5) owing to the structural similarity in H antigens, namely serovars Virchow (6,7,14:r:1,2), Bareilly (6,7,14:y:1,5), Thompson (6,7,14:k:1,5), and Choleraesuis (6,7:c:1,5). A circular plot of the pangenome of these five serovars (Infantis: 13 strains, Virchow: 4 strains, Bareilly: 27 strains, Thompson: 9 strains, and Choleraesuis: 3 strains) revealed that the *usg* gene and its homologues were conserved only in serovar Infantis (Figure 1B).

### 3.2. In Silico Prediction of Sensitivity and Specificity of the Candidate Gene Marker

**Figure 2:**
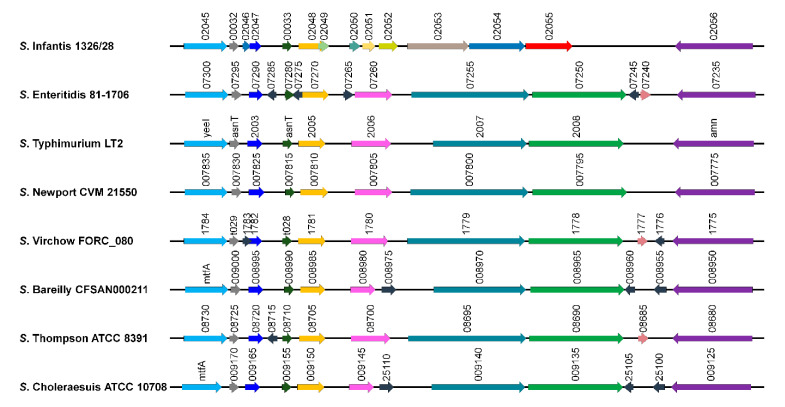
Comparison of *usg* loci among eight serovars (Infantis, Virchow, Bareilly, Thompson, Choleraesuis, Enteritidis, Typhimurium, and Newport). A consistent color coding indicates orthologous genes. The locus of the *usg* gene is highlighted in red.

The loci containing the *usg* gene or its homologues were compared in detail between eight *Salmonella* strains representing each serovar, including five (Infantis, Virchow, Bareilly, Thompson, and Choleraesuis) with similar antigenic determinants, and three (Enteritidis, Typhimurium, and Newport) known for high prevalence (Figure 2). The locus encompassing approximately 9541 bp from SIN_02045 to SIN_02056 in *S.* Infantis strain 1326/28 genome was absent in the other seven genomes, and was replaced with ~10,423 bp sequences showing high sequence homology among the other serovars. This finding implies a foreign-DNA acquisition event in serovar Infantis during evolutionary divergence of *Salmonella* serovars. The *usg* gene is predicted to encode a hypothetical protein with a SIR2-like domain. Silent information regulator 2 (SIR2) enzymes catalyze NAD^+^-dependent protein/histone deacetylation and are found in diverse organisms, including eukaryotes, archaea, and prokaryotes. The conserved SIR2-like domain of the Usg protein is also observed in *Cronobacter sakazakii*, *Klebsiella pneumoniae*, and *Enterobacter* spp. in the Enterobacteriaceae family.

**Figure 3:**
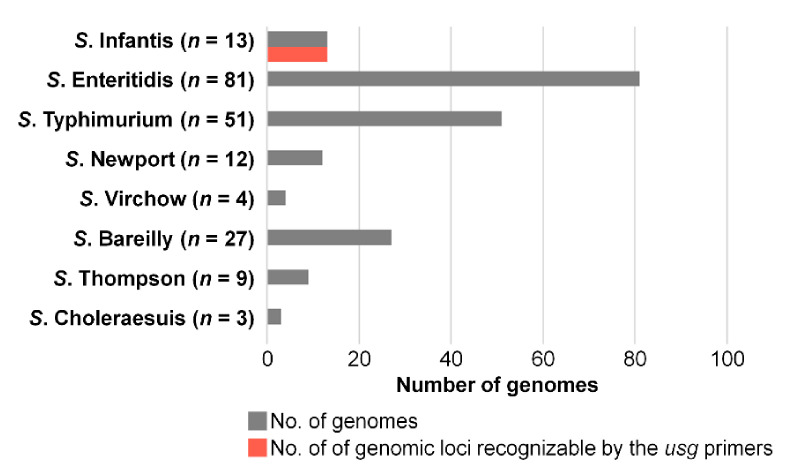
Evaluation of genotyping efficacy in silico. Genomic loci recognizable by the proposed diagnostic PCR primer set were counted in eight *Salmonella* serovars. The number of genomes analyzed in each serovar is shown in the gray bar, and the number of genomic loci that could be amplified by the diagnostic primers is shown in the red bar.

A primer set that specifically binds to *usg* or its homologous genes was designed using Primer Designer to obtain a 109 bp PCR product, and its genoserotyping efficacy was evaluated in silico using 568 genome sequences of 60 serovars. The primer set was expected to successfully discriminate Infantis from other serovars. When the eight serovars were examined, it was predicted that the primer set would recognize all Infantis genomes of 13 strains but not bind to 187 genomes of the other seven serovars (Enteritidis, *n* = 81; Typhimurium, *n* = 51; Newport, *n* = 12; Virchow, *n* = 4; Bareilly, *n* = 27; Thompson, *n* = 9; and Choleraesuis, *n* = 3; Figure 3). Sensitivity, defined as a positive rate against Infantis genomes, was 100%, and specificity, defined as a negative rate against non-Infantis genomes from the other seven serovars, was 100%.

### 3.3. Efficacy Evaluation of the Diagnostic Primer Set in Real-Time PCR

**Figure 4:**
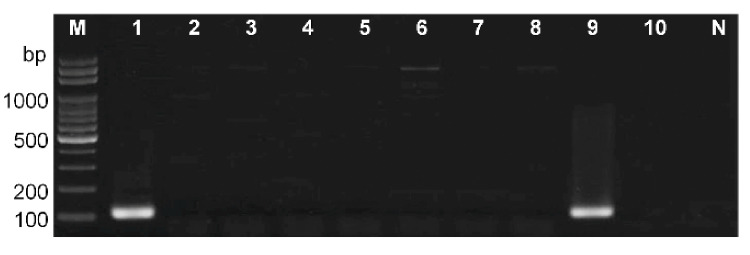
PCR discrimination of Infantis from seven other serovars by means of the proposed diagnostic primer set. PCR with the *usg*-specific primers was applied to *S*. Infantis strain CCARM 8283 (lane 1), *S*. Enteritidis MFDS 1010897 (lane 2), *S*. Typhimurium ATCC 19585 (lane 3), *S*. Newport FORC 020 (lane 4), *S*. Virchow MFDS 1004870 (lane 5), *S*. Bareilly MFDS 1007637 (lane 6), *S*. Thompson CCARM 8530 (lane 7), *S*. Choleraesuis ATCC 13312 (lane 8), a mixture of the eight serovars (lane 9), and a mixture of the seven serovars other than *S*. Infantis (lane 10). Lanes M and N correspond to a 100 bp DNA ladder and a negative control lacking a DNA template, respectively.

The diagnostic primer set for the Infantis *usg* gene amplified a 109 bp DNA fragment in PCR analysis of pure culture of *S.* Infantis strain CCARM 8283, and did not yield any equivocal amplicon in the PCR analysis of pure cultures of the other seven serovars (Figure 4). This primer set also successfully amplified the fragment of the Infantis *usg* gene in a mixture of eight serovars, suggesting the feasibility of PCR-based Infantis identification in mixed *Salmonella* cultures prepared from contaminated environmental samples.

**Figure 5:**
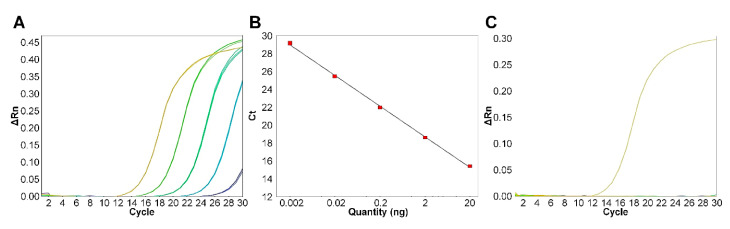
Performance of the diagnostic primer set in real-time PCR. (**A**) Amplification plots obtained from serial dilutions of *S*. Infantis CCARM 8283 genomic DNA from 20 ng to 2 pg: yellow, 20 ng; yellow-green, 2 ng; green, 200 pg; cyan, 20 pg; and blue, 2 pg. (**B**) The standard curve of dependence of C_t_ values (*Y*) on genomic-DNA amounts (*X*) corresponded to the equation *Y* = −3.42 *X* + 19.983 (R^2^ = 0.999). (**C**) Specificity of the primer set in relation to 60 *Salmonella* strains. None of the strains yielded amplification, except *S*. Infantis CCARM 8283. Strains tested in (**C**) are listed in Supplementary Table S2.

The genomic DNA of *S.* Infantis CCARM 8283 was serially diluted 10-fold from 20 ng to 2 pg, and was employed as a template in real-time PCR with the *usg*-specific primer set. Threshold cycles (C_t_) ranged from 15 (20 ng) to 29 (2 pg), showing strong dependence on the tested DNA amounts (Figure 5A). When the relationship between C_t_ values and DNA template amounts was plotted, an absolute linear plot was obtained with an R^2^ value of 0.999 and 95.76% efficiency (Figure 5B). The stepwise decrease in C_t_ values in proportion to DNA amounts is indicative of the suitability of bacterial quantification by real-time PCR. Equivalent DNA amounts (20 ng) from 60 *Salmonella* strains representing all the studied serovars were subjected to real-time PCR with the *usg*-specific primer set. All serovars except *S.* Infantis failed PCR amplification, indicating high specificity of the *usg*-specific primer set to serovar Infantis (Figure 5C).

### 3.4. Validation of the Diagnostic PCR Method

**Figure 6:**
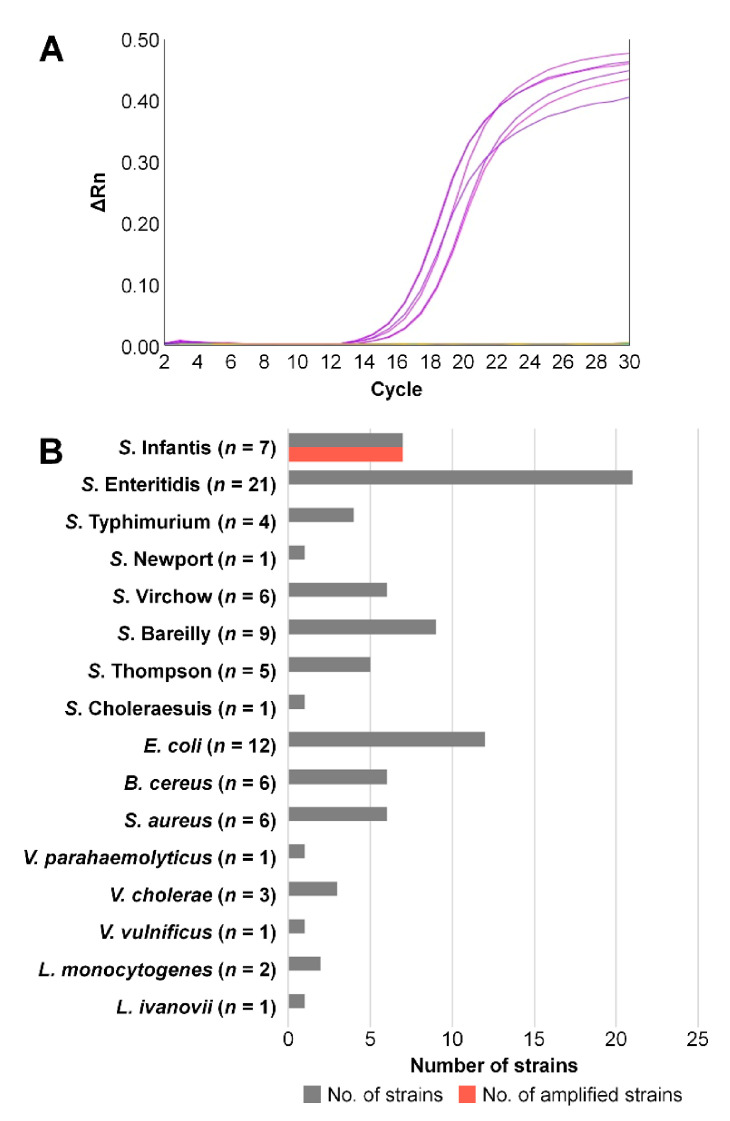
Validation of the specificity of the designed primer set using isolates of diverse origins. (**A**) Real-time PCR results on 54 *Salmonella* strains. Only the seven tested *S.* Infantis strains yielded a PCR product. (**B**) Specificity results on 54 *Salmonella* strains and 32 non-*Salmonella* strains. Gray bars denote the numbers of tested strains, and red bars represent the numbers of strains that yielded amplification with the proposed diagnostic primer set.

The sensitivity and specificity of the devised diagnostic assay were evaluated on 54 *Salmonella* strains (Infantis, *n* = 7; non-Infantis, *n* = 47) and 32 non-*Salmonella* pathogenic strains (pathogenic *Escherichia coli*, *n* = 12; *Vibrio* spp., *n* = 5; *Listeria monocytogenes*, *n* = 2; *L. ivanovii*, *n* = 1; *Staphylococcus aureus*, *n* = 6; and *Bacillus cereus*, *n* = 6). The *Salmonella* strains, except serovar Infantis, included three frequently isolated serovars (Enteritidis, *n* = 21; Typhimurium, *n* = 4; and Newport, *n* = 1) and four serovars with similar H antigenic structures that were therefore likely to yield false positive results (Virchow, *n* = 6; Bareilly, *n* = 9; Thompson, *n* = 5; and Choleraesuis, *n* = 1). Detailed information about the bacterial strains is given in Supplementary Table S2. Bacterial serovars were confirmed by conventional serotyping through agglutination of antisera. All the tested Infantis strains yielded amplicons with C_t_ values between 14.5 and 17.4 (Figure 6A), showing 100% sensitivity. In contrast, the diagnostic primer set did not give any amplification for the 47 non-Infantis strains and 32 non-*Salmonella* strains, thus manifesting 100% specificity (Figure 6B). This testing of diverse bacterial species and strains of various origins validated the diagnostic primer set as being suitable for rapid and accurate identification of *Salmonella* Infantis.

## 4. Discussion

*Salmonella* spp. are a leading cause of bacterial gastroenteritis globally, and the main infection sources are frequently linked with poultry and eggs. For several decades, two host-nonspecific serovars, *S.* Enteritidis and *S.* Typhimurium, have been dominant among foodborne infection cases. Nonetheless, the prevalence of serovar Infantis has been increasingly apparent since the late 1970s, and this serovar ranks among the top 10 human isolates of *Salmonella* spp. worldwide [5,6,7]. This striking change in serological predominance is attributable to the high prevalence of *S.* Infantis in broiler and layer flocks. Vaccination against serovars Enteritidis and Typhimurium, the most often reported serovars of human *Salmonella* infections, has been performed on poultry farms in many countries. Live vaccines derived from serovars Enteritidis and Typhimurium may trigger immunity against these two serovars in poultry; however, the aggressive eradication implemented against these two serovars may have accidently led to the emergence of serovar Infantis, replacing the above-mentioned serovars in the same ecological niche [5,8]. It has been observed that *S.* Enteritidis became prominent in poultry farming after the eradication of *S.* Gallinarum in the 20th century [27]. As the risk of *S.* Infantis infections is expected to increase further, thorough surveillance is critical for controlling *S.* Infantis.

Owing to the drawbacks of conventional culture-based serotyping methods, which are laborious and time-consuming, molecular methods have been developed for the identification of common serovars. Among these techniques, PCR-based assays continue to gain ground because of their simple and rapid identification of pathogens. A number of primers and probes have been developed to differentiate between notorious serovars *S.* Enteritidis and *S.* Typhimurium [28,29,30]. There have also been several reports offering PCR-based molecular diagnostics for *S.* Infantis. One research group designed PCR primers specific to serovar Infantis by finding conserved sequences in H-antigen-determinant genes *fljB* and *fliC* of five *S.* Infantis strains [8]. More recently, multiple cross-displacement amplification methods employing multiple primer sets were combined with isothermal polymerization reaction, and thereby enhanced the sensitivity and rapidity of PCR-based Infantis diagnostics [18]. In addition, chromogenic biochip methods have been incorporated into practice to improve onsite application of PCR-based diagnostics [31]. Nonetheless, the current molecular serotyping methods targeting serovar-specific DNA markers occasionally fail to show high resolution performance because these methods exploit only a subset of genes in the genome. In this context, multilocus sequence typing methods that discriminate among multiple genetic loci have been proposed to increase genoserotyping resolution [19]. Despite these apparent advances in molecular diagnostics using DNA markers, the sensitivity, specificity, and accuracy of these methods are still not equivalent to the performance of classic serotyping methods. Fortunately, the availability of whole-genome sequences of *Salmonella* spp. is expanding almost daily, and more specifically designed primers and probes can help to delineate serological differences among *Salmonella* serovars.

Our diagnostic marker gene (*usg*) specific to serovar Infantis was identified via the pangenome profiling of 568 *Salmonella* spp. strains from 60 dominant serovars. The sensitivity and specificity of diagnostic methods can be improved when as many *Salmonella* whole-genome sequences as possible are input into the comparative genomic analysis for identifying the core genome retained in serovar Infantis and the pangenome covering all non-Infantis serovars. In comparison with the previously devised molecular methods, we analyzed a greater number whole-genome sequences from Infantis and non-Infantis strains to find a gene marker specific to serovar Infantis; thus, the sensitivity and accuracy of our diagnostic method are much higher [18,19]. The deduced gene markers are sometimes absent in newly isolated strains when a small number of genome sequences are used in comparative genomics [18]. Considering the steep increase in serovar Infantis prevalence among human and food animal isolates, the devised genoserotyping assay represents a sensitive, rapid, and convenient surveillance tool as an alternative to the conventional serotyping method. Furthermore, the proposed technique does merit integration with existing chromogenic devices for onsite application or adaptation to multiplex PCR assays for serotyping of prevalent serovars.

## Data Availability

The data presented in this study are available on request from the corresponding author.

## Supplementary Materials

The following are available online at https://www.mdpi.com/2076-2607/9/1/67/s1, Figure S1: Pangenome distribution of 13 *S*. Infantis genomes, Table S1: Genome features of 568 *Salmonella* stains, Table S2: Bacterial strains used in this study.
