# Supplementary material for: Development of a Genoserotyping Method for Salmonella Infantis Detection on the Basis of Pangenome Analysis
Source: Microorganisms. 2020 Dec 29;9(1):67. doi: 10.3390/microorganisms9010067 (PMC7824266; doi:10.3390/microorganisms9010067)
Supplement: Supplementary file 1 [file microorganisms-09-00067-s001.zip › Supplementary Figure S1.pdf]

Supplementary Information

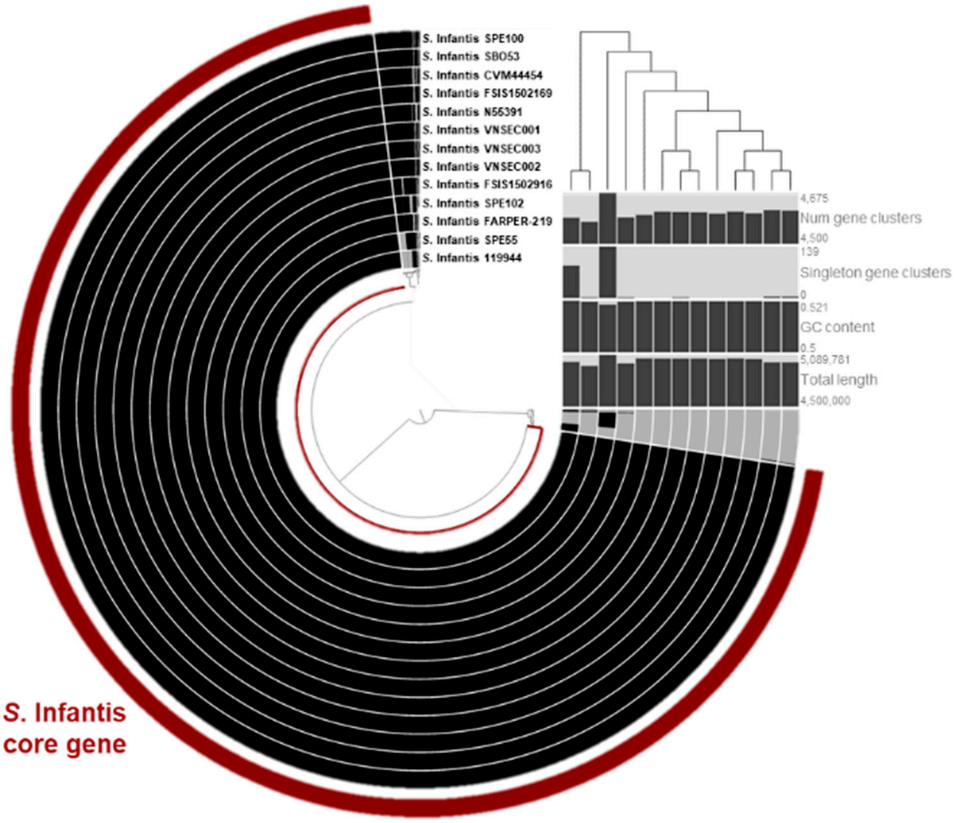

Figure S1. Pan-genome distribution of 13 *S. Infantis* genomes. Black rings represent *S. Infantis* genomes, and a red arc represents the core gene region conserved among *S. Infantis* genomes.
